# Intensity-Modulated Radiation Therapy Versus 3D Conformal Radiotherapy for Postoperative Gynecologic Cancer: Are They Covering the Same Planning Target Volume?

**DOI:** 10.7759/cureus.467

**Published:** 2016-01-25

**Authors:** Jelena Lukovic, Nikhilesh Patil, David D'souza, Barbara Millman, Brian P Yaremko, Eric Leung, Frances Whiston, George Hajdok, Eugene Wong

**Affiliations:** 1 Department of Radiation Oncology, London Regional Cancer Program, London, Ontario, CA; 2 Schulich School of Medicine & Dentistry, Western University, London, Ontario, CA; 3 Nova Scotia Cancer Centre, Nova Scotia Health Authority, Dalhousie University; 4 Department of Radiation Oncology, London Regional Cancer Program, London, Ontario, CA; 5 Department of Physics and Engineering, London Health Sciences Center, London, Ontario, CA; 6 Toronto Sunnybrook Hospital, University of Toronto; 7 Clinical Research Unit, London Health Sciences Center, London, Ontario, Canada; 8 Division of Radiation Oncology, London Regional Cancer Program, London, Ontario, CA

**Keywords:** intensity-modulated radiotherapy (imrt), gynecological cancers, cervix cancer, endometrial cancer, dosimetry

## Abstract

Background and Purpose: This study compares dosimetric parameters of planning target volume (PTV) coverage and organs at risk (OAR) sparing when postoperative radiotherapy for gynecologic cancers is delivered using volumetric modulated arc therapy (VMAT) versus a four-field (4FLD) box technique.

Material and Methods: From July to December 2012, women requiring postoperative radiation for gynecologic cancers were treated with a standardized VMAT protocol. Two sets of optimized 4FLD plans were retrospectively generated: one based on standard anatomical borders (4FLD) and one based on the clinical target volume (CTV) created for VMAT with a 2 cm expansion guiding field border placement (4FLD+2). Ninety-five percent isodose curves were generated to evaluate PTV coverage.

Results: VMAT significantly improved dose conformity compared with 4FLD and 4FLD+2 plans (p < 0.001) and provided additional coverage of the PTV posteriorly and superiorly, corresponding to coverage of the presacral and proximal iliac vessels. There was a significant reduction in dose to all OARs with VMAT, including a 58% reduction in the volume of the small bowel receiving more than 45 Gy (p=0.005).

Conclusions: Despite treating a larger volume, radiotherapy using a 4FLD technique is less homogenous and provides inferior coverage of the PTV compared with VMAT. With meticulous treatment planning and delivery, VMAT effectively encompasses the PTV and minimizes dose to OARs.

## Introduction

The role of adjuvant radiation in the treatment of endometrial and cervical malignancies has been well established with a number of randomized clinical trials [1-5]. Postoperative pelvic radiotherapy has been shown to improve pelvic control for uterine cancer in patients with intermediate or high-risk pathologic features, including high tumor grade, lymphovascular space invasion, and deep myometrial invasion [1, 3-5]. Similarly, for cervix cancer, postoperative pelvic radiotherapy improves locoregional control and overall survival in patients with high-risk features [6-7].

Pelvic radiotherapy has been classically delivered with three-dimensional conformal radiation therapy (3DCRT) using a four-field (4FLD) box technique based on standard anatomical borders. It is generally assumed that 3DCRT provides similar coverage of the planning target volume (PTV) generated for intensity-modulated radiotherapy (IMRT) with less chance of geographic miss. Postoperatively, a portion of the gastrointestinal tract falls into the pelvic cavity and is exposed to the prescribed dose, leading to clinically significant toxicity and limiting the safely deliverable dose to 45 Gy to 50 Gy [8]. In the Post-Operative Radiation Therapy in Endometrial Carcinoma 1 (PORTEC-1) Trial, 26% of patients reported limitations of daily activity related to bowel symptoms [1, 9]. In this trial, 52% of patients were treated using a four-field technique and their cumulative five-year complication rate was 21% [10]. In patients receiving chemotherapy in addition to radiation, a 50% risk of acute Grade 4 small bowel toxicity and a 34% risk of late toxicity have been reported [11]. Since a significant portion of the total body bone marrow reserve is located within the lower lumbar spine and pelvic bones, standard 4FLD pelvic radiotherapy fields include a substantial volume of bone marrow resulting in the depletion of hematopoietic stem cells and hematologic toxicity. Resultantly, the use of chemotherapy regimens that rely on bone marrow reserve may be limited [12].

IMRT has been explored as a mechanism to reduce the dose to organs at risk (OAR) while maintaining dose to the clinical target volume (CTV). Several dosimetric studies have retrospectively evaluated organs at risk sparing using IMRT versus conventional pelvic radiotherapy and demonstrated statistically significant reductions in the volume of small bowel, bladder, rectum, and bone marrow receiving the prescription dose [13-14]. For example, Beriwal, et al. evaluated clinical outcomes of patients treated with IMRT in the postoperative setting. With a median follow-up of 20 months, they demonstrated 100% local control and a 2.1% rate of Grade 2 or higher chronic toxicity at three years [15].

In July 2012, our institution began treating all postoperative patients with endometrial or cervical cancer according to a standardized protocol using volumetric modulated arc therapy (VMAT). It was appreciated that an anatomic-based system of generating target volumes for VMAT was different from 3DCRT where it is assumed that bony landmarks with some modification adequately define the fields to cover the volume at risk. We sought to compare how well 3DCRT covers the PTV generated for IMRT-based treatment. Hence, the purpose of this study was to compare dosimetric parameters of PTV coverage and OAR sparing when postoperative radiotherapy for endometrial and cervical cancer is delivered using VMAT versus 3DCRT using a classic 4FLD box technique.

The Lawson Health Research Institute Research Ethics Board approved this protocol (#103185). Informed patient consent was obtained at the time of treatment.

## Materials and methods

From July to December 2012, all women with endometrial or cervical cancer receiving postoperative radiation were treated with a standardized VMAT protocol. Radio-opaque fiducial markers were placed in the vaginal vault apex at the time of radiation planning. CT simulation was performed with a full and empty bladder.

OARs were contoured on the full bladder scan using RTOG guidelines and included bladder, bowel cavity, rectum, femoral heads, and bone marrow (entire pelvic bones) [16]. The empty and full bladder scans were used to create the vaginal internal target volume (ITV). The nodal clinical target volume (CTV) was contoured to include the obturator, internal iliac, external iliac, and common iliac lymph nodes by adding a 7 mm margin on vessels, adjacent nodal tissue, lymphoceles, and surgical clips, as outlined in consensus guidelines [17]. Presacral lymph nodes were contoured from S1 to the bottom of S2, depending upon specific disease characteristics. A 10 mm expansion was used to generate the nodal and vaginal PTVs. During treatment, daily cone-beam CT imaging was performed to ensure that target volumes were encompassed within the PTV. VMAT planning was performed using SmartArc in Pinnacle, version 9.6 (Philips Healthcare USA). Two 6 MV 360-degree arcs were used. Field sizes and collimator angles were optimized for target coverage, OAR sparing, and reduction of interleaf leakage throughout the arc range. A priority during optimization was placed on PTV coverage while reducing the dose to OARs, adhering to standardized dose limits.

The prescription dose was 45.0 Gy to 50.4 Gy in 1.8 Gy fractions to the PTV. Treatment plans were generated so that that 99% of the prescription isodose surface would encompass ≥ 90% of the PTV, ≥ 99% of the PTV would receive ≥ 90% of the prescription dose, ≥ 97% of the PTV would receive ≥ 97% of the prescription dose, < 1% of PTV would receive ≥ 115% of the prescription dose, < 10% of the PTV would receive ≥ 110% of the prescription dose, and that the dose maximum occurs within the PTV. Table 1 outlines the normal tissue dose constraints.

**Table 1:** Organ at Risk Dose Constraints.

Tissue	Limits		Maximum Dose
Bowel Cavity	V_45_ ≤ 200 cc	V_40 _< 30%	< 50Gy
Rectum	V_45_ < 50%	V_30 _< 60%;	< 50Gy
Bone Marrow	V_10_ < 80%	V_20_ < 66%	
Bladder	V_45_ < 50%		< 50Gy
Femoral Head	V_30 _< 15%		< 50Gy

Two sets of optimized 3DCRT plans were retrospectively generated. 4FLD plans were based on anatomical borders, which were used by our institution prior to the introduction of VMAT. These borders are:  Superior - L5/S1; Inferior - Bottom of the obturator foramen or at least 3 cm below fiducial markers; Lateral - 2 cm on the pelvic brim, with adjustments based on vessel contours; Anterior - 5 mm anterior to pubic symphysis with adjustments based on vessel contours; and Posterior - S2/S3. The second set of plans was generated using the CTV created for VMAT to guide field border placement as is done at some centers; for these plans, referred to as 4FLD+2, a 2 cm expansion on the CTV was used to guide placement of the field borders and ensure adequate PTV coverage. 

Ninety-five percent isodose curves were generated for the three sets of plans (4FLD, 4FLD+2, and VMAT) to evaluate PTV coverage. Areas of overlap were quantified and regions covered by one technique but not the others were qualitatively assessed to determine patterns of missed coverage. Dose-volume histograms for the PTV and OARs were generated for the treatment plans and compared using analysis of variance (ANOVA) and pairwise t-tests.

This retrospective study was approved by our local Research Ethics Board.

## Results

Twenty patients, aged 37 to 88, received 45 Gy to 50.4 Gy in 1.8 Gy fractions, five fractions per week. Patient characteristics are summarized in Table 2.

**Table 2:** Patient Characteristics.

	Cervix	Endometrium
Chemotherapy	3	6
Histology		
Adenocarcinoma	8	3
Adenosquamous	1	1
Endometrioid	3	
Serous	1	
Leiomyosarcoma	1	
Carcinosarcoma	2	
Grade		
1	4	1
2	7	1
3	1	
Not Reported	4	2
FIGO Stage		
I	7	2
II	2	
III	7	1
IVA		1
IVB		
Brachytherapy Boost	12	

**Figure 1:**
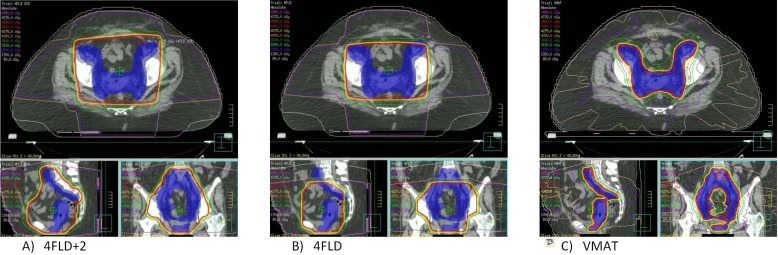
Comparative dose distributions for A) 4FLD+2, B) 4FLD, and C) VMAT.

Comparative dose distributions obtained with 4FLD, 4FLD+2, and VMAT plans are illustrated in Figure 1.

The mean conformity index (CI = prescription isodose volume/target (PTV) volume) was 1.99 + 0.5 with 4FLD, 1.73 + 0.46 with 4FLD+2, and 0.81 ± 0.13 with VMAT; VMAT planning significantly improved dose conformity compared with both 4-field plans (p < 0.001). Dose conformity with 4FLD and 4FLD+2 plans were not significantly different (p=0.07). The homogeneity indices were calculated as HI = (D_2_-D_98_)/D_p_, where D_2_ is the minimum dose to 2% of the target volume, D_98_ is the minimum dose to the 98% of the target volume, and D_p_ is the prescribed dose. The mean homogeneity indices were: 0.97 ± 0.12 (4FLD), 0.55 ± 2.05 (4FLD+2), and 0.07 ± 0.02 (VMAT).

There was a significant reduction in dose to all OARs with VMAT plans compared with 4FLD and 4FLD+2 plans, including a 58% mean reduction in the volume of small bowel receiving more than 45 Gy (p=0.005).

**Figure 2:**
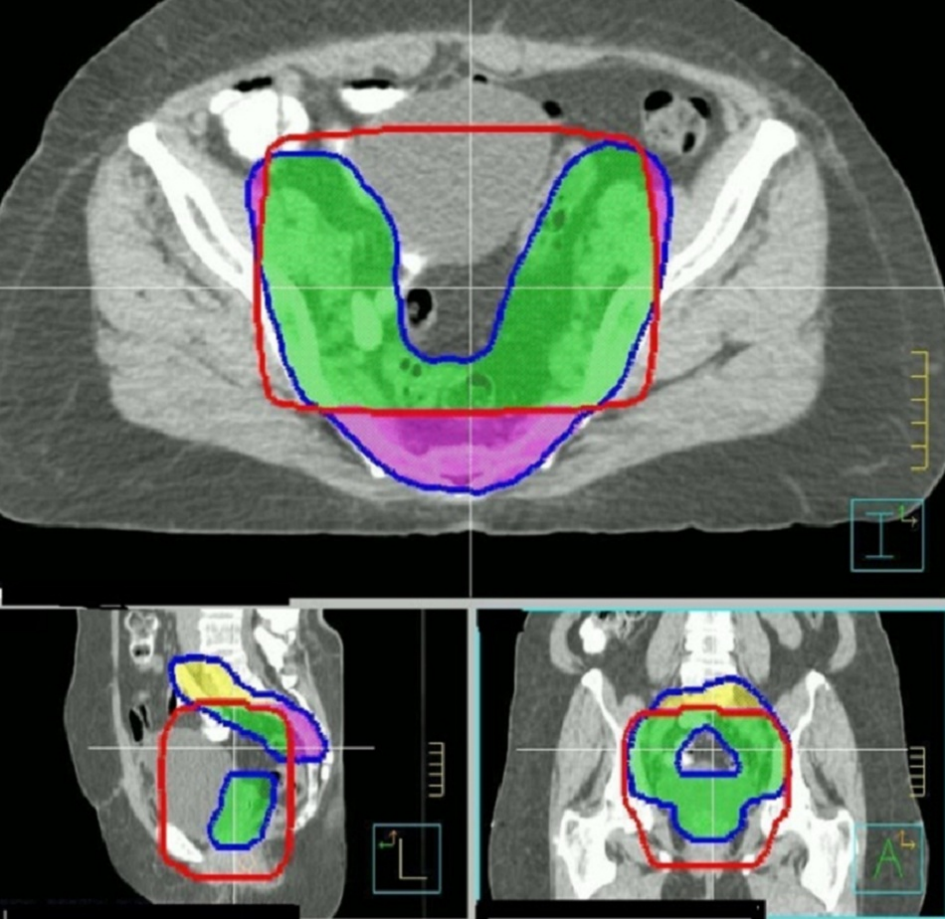
PTV coverage with a 4FLD plan generated based on standard anatomic borders. Undercovered areas are posterior (purple) and superior (yellow).

4FLD plans consistently undercovered posterior and superior aspects of the PTV (Figure 2).

On average, VMAT plans covered an additional 75.9 ± 50.9 cc posteriorly and 236.9 ± 189.5 cc superiorly; these areas correspond to coverage of the presacral and proximal iliac vessels respectively. PTV coverage improved with 4FLD+2 plans; however, this was at the expense of a significantly increased the dose to all OARs (p < 0.001) (Table 3).

**Table 3:** Dosimetric Parameters for OARs for 4FLD, 4FLD+2, and VMAT Plans

		4FLD	4FLD+2	VMAT
Bladder	V45%	95.4±6.2	90.9±16.8	19.9±11.7
Rectum	V30%	95.5±5.5	91.5±8.6	66.3±19.2
	V45%	63.8±19.8	53.8±28.2	19.7±17.6
Small Bowel	V40%	35.74±22.1	43.3±21.9	20.9±11.6
	V45(cc)	387.8±194.8	461.9±177.9	121.2±74.0
Bone Marrow	V10%	84.3±10.1	93.0±3.7	81.1±5.8
	V20%	80.1±5.4	87.5±5.5	64.5±6.7
Femoral Head (Left)	V45%	4.9±3.6	7.2±8.3	0.08±0.23
Femoral Head (Right)	V45%	6.4±4.1	6.8±8.0	0.08±0.21

## Discussion

Adjuvant pelvic radiotherapy is recommended for patients with intermediate or high-risk endometrial cancer and for patients with high-risk cervical cancer because it significantly improves local control (20% vs. 5%, p < 0.001) [1, 9]. The improvement in locoregional control, however, is at the expense of increased acute and late gastrointestinal and genitourinary toxicity [9-11]. Current literature suggests that 60% of women experience acute Grade 1 to 2 gastrointestinal toxicity following EBRT with 20% continuing to experience symptoms at five years [9, 18]. Severe Grade 3 to 4 complications are seen in 3% of patients [9]. Further, a substantial amount of bone marrow is treated in standard 4FLD pelvic radiotherapy leading to hematologic toxicity; this may be further exacerbated with the use of concurrent chemotherapy [12].

Intensity-modulated radiotherapy (IMRT) uses beams of varying intensity to deliver high doses of radiation to complex geometrical targets while constraining dose to healthy tissue, theoretically decreasing late effects [19]. Mundt, et al.explored the use of IMRT without concurrent chemotherapy for postoperative gynecologic cancers and reported a significant reduction in overall toxicity with no Grade 3 or higher toxicities [20]. Compared with 4FLD plans, IMRT was associated with a significant reduction in Grade 2 acute gastrointestinal toxicity (36% vs. 80%) as well as a 30% reduction in chronic gastrointestinal toxicity [20]. IMRT also has the potential to minimize dose to bone marrow, which becomes particularly important if considering more intensive concurrent and adjuvant chemotherapy regimens [12]. The main benefit reported thus far from the use of IMRT compared with 4FLD radiotherapy has been a reduction in toxicity.

We focused on comparing how well 4FLD plans cover the PTV developed for use with IMRT. We assumed that most radiation oncologists who use 3DCRT treat with similar techniques and field borders. Since initial analyses showed that these plans would not adequately cover the CTV used for IMRT, we added an additional comparison with 2 cm around the CTV, guiding field border placement (4FLD+2). Resultantly, we evaluated three methods of pelvic radiotherapy treatment planning for postoperative gynecologic cancers: VMAT plans created using CT-based anatomy to delineate the CTV and OAR contours, 4FLD plans generated based on standard anatomical borders with adjustments based on vessel contours, and 4FLD+2 plans generated using a 2 cm expansion on the same CTV used for VMAT plans to guide field placement. As previously reported, we observed a statistically significant reduction in the irradiated volume of all OARs with the VMAT plans [11-15, 21-23].

Our study confirms many of the expected advantages of VMAT, such as improved dose conformity and homogeneity. We also demonstrate that PTV coverage with VMAT is superior to 4FLD plans and that generating treatment plans using bony anatomy alone may be suboptimal. When treatment plans are generated using a 2 cm expansion on the VMAT CTV to guide field border placement, PTV coverage improves; however, the dosimetric improvement in PTV coverage is at the expense of increased OAR dose.

There are several reasons for these discrepancies. The original 4FLD technique was based on a “one size fits all” approach that was used to guide treatment. Subsequently, it was shown that using standard bony landmarks alone provided inadequate coverage of lymph nodes as defined on lymphangiograms for cervix cancer [24]. Lymphangiography fell with the advent of CT imaging where detailed work was done to map lymph nodes in relation to vessels [25-27]. The development of consensus guidelines for contouring nodal CTVs based on vessel anatomy has been generated from such work [17, 24]. Based on this experience, a 7 mm expansion around representative vessels was used to generate the nodal CTV.

We generated 4FLD borders and shielding based on vessel position as was done prior to the implementation of VMAT. This was done by contouring the vessels and ensuring that the field border was 1.5 cm to 2 cm beyond them rather than using bony landmarks, such as the pelvic brim, alone. This process is inherently different from one where a nodal CTV, which is larger than vessels alone, is generated and an additional margin is added to create the PTV. In our prior experience with 4FLD planning, vessel contours guide the field borders but are not formally evaluated for coverage as a CTV. Given the GI toxicity that ensues from postoperative pelvic radiotherapy, we suspect that there is a tendency to be tight with field borders to reduce the amount of bowel radiated. This motivates the question: how important is it that the consensus derived CTV is covered in the delivery of external beam radiation? Our data suggest that in addition to treating OARs to a higher dose, 3DCRT does not provide good coverage of the CTV unless wide fields are used. Regardless, the results reported in the era of 3DCRT have been generally good [1, 5-7, 22].

Our study also demonstrates that the areas where VMAT plans provide additional PTV coverage compared with 4FLD plans are the posterior and superior aspects. Superiorly, contouring the common iliac vessels shifts the superior aspect of the field higher than L5/S1. In our prior clinical experience, without the ability to limit the volume of bowel treated, extending the superior border above L5/S1 may increase chronic GI toxicity. Posteriorly, the undercoverage of the PTV correlates with the presacral region; this area is simply not covered with a “standard” posterior border on the lateral field placed at S2/3. It is unclear whether better coverage of the PTV, as seen in our study, will translate into lower recurrence rates. With CT-based simulation and planning, some centers formally define a CTV and use a 2 cm expansion to guide field border placement. When plans were generated using this technique (4FLD+2), we were able to compensate for the poorer PTV coverage, but at the expense of increased OAR dose.

Our study demonstrates that the coverage of what is felt by consensus opinion to be appropriate for postoperative gynecologic cancers is covered at least as well if not better by VMAT compared to 3DCRT using 4FLD techniques. If 3DCRT is used, a 2 cm expansion on the CTV may also provide adequate target volume coverage. RTOG1203, a randomized clinical trial comparing the 4FLD technique to IMRT will provide additional data in this regard.

Concerns have been raised that the high-precision planning and intended radiation delivery of VMAT may come at the expense of compromised coverage of the PTV secondary to target motion. Some argue that the tight conformity realized by VMAT may adversely affect oncologic outcomes secondary to undertreatment of the PTV [7-8]. Delivery of VMAT for this cohort of women was done using daily localization with cone-beam CT imaging with soft tissue match and fiducial marker alignment. This aspect of using a robust method of treatment verification is of critical importance to ensure that the PTV is treated as intended.

There are limitations that preclude widespread implementation of VMAT and more data are required to optimally define its advantages. Patient immobilization is essential for daily reproducibility, and adequately controlling for variations in daily bladder filling and bowel distention throughout treatment are important; they remain challenging at this time. Studies investigating the effects of internal organ motion, particularly relating to the effects of bladder and rectal filling, are essential in the creation of consensus guidelines for the delineation of VMAT volumes so that the use of this technique can be maximized in this cohort. This trial provides parameters suggesting reduced toxicity risk, but clinical data are not available for validation. Furthermore, given that inappropriate delineation of targets may result in tumor recurrence, careful assessment of clinical outcomes is warranted.

## Conclusions

Despite treating a larger overall volume, 3DCRT delivered using a 4FLD plan compared to VMAT is less homogenous and provides inferior coverage of the PTV. Three-dimensional conformal radiation therapy delivered using a 4FLD+2 plan, however, provides similar PTV coverage to VMAT at the expense of increased OAR doses. With meticulous treatment planning and delivery, VMAT offers a more effective method of delivering radiation to encompass the PTV and minimize spillage of dose to surrounding tissue. Furthermore, by allowing for conformation of dose to target shape, VMAT minimizes OAR high-dose volumes.

In summary, we demonstrated the dosimetric superiority, with respect to PTV coverage and OAR sparing, of VMAT over 4FLD and 4FLD+2 plans in the adjuvant treatment of gynecologic malignancies, although concerns over internal organ motion remain. Further studies investigating the effects of internal organ motion and creation of consensus guidelines for VMAT target delineation are essential in standardizing the use of this technique in this cohort. 
